# Crystal structure and Hirshfeld surface analysis of *N*-(*tert*-but­yl)-2-(phenyl­ethyn­yl)imidazo[1,2-*a*]pyridin-3-amine

**DOI:** 10.1107/S2056989019012751

**Published:** 2019-09-27

**Authors:** Zahira Tber, Sevgi Kansiz, Mohamed El Hafi, Mohamed Loubidi, Jabrane Jouha, Necmi Dege, El Mokhtar Essassi, Joel T. Mague

**Affiliations:** a Laboratoire de Chimie Bioorganique & Analytique, URAC 22 Université Hassan II Mohammedia-Casablanca, Faculté des Sciences et Techniques, BP 146, 28800 Mohammedia, Morocco; b Ondokuz Mayıs University, Faculty of Arts and Sciences, Department of Physics, 55139 Samsun, Turkey; c Laboratoire de Chimie Organique Hétérocyclique, Centre de Recherche des Sciences des Médicaments, Pôle de Compétence Pharmacochimie, Av Ibn Battouta, BP 1014, Faculté des Sciences, Université Mohammed V, Rabat, Morocco; d Laboratoire de Chimie Organique et Analytique, Université Sultan Moulay Slimane, Faculté des Sciences et Techniques, BP 523, 23000 Beni-Mellal, Morocco; e Ondokuz Mayıs University, Faculty of Arts and Sciences, Department of Physics, 55139, Samsun, Turkey; f Department of Chemistry, Tulane University, New Orleans, LA 70118, USA

**Keywords:** crystal structure, alkyne, imidazo[1,2-*a*]pyridin, hydrogen bonding, C—H⋯π(ring) inter­action, Hirshfeld surface

## Abstract

The bicyclic imidazo[1,2-*a*]pyridine core of the title compound, C_19_H_19_N_3_, is relatively planar with an r.m.s. deviation of 0.040 Å. The phenyl ring is inclined to the mean plane of the imidazo[1,2-*a*]pyridine unit by 18.2 (1)°. In the crystal, mol­ecules are linked by N—H⋯H hydrogen bonds, forming chains along the *c*-axis direction. The chains are linked by C—H⋯π inter­actions, forming slabs parallel to the *ac* plane. The Hirshfeld surface analysis and fingerprint plots reveal that the crystal structure is dominated by H⋯H (54%) and C⋯H/H⋯C (35.6%) contacts. The crystal studied was refined as an inversion twin

## Abstract 2

In the title compound, the phenyl ring of the phenyl-ethynyl substituent is inclined to the mean plane of the imidazo[1,2-*a*]pyridine moiety by 18.2 (1)°.

## Chemical context   

Compounds containing the imidazo[1,2-*a*]pyridine moiety have received considerable attention because of their inter­esting biological activities. For instance, it is found in several commercialized drugs such as the sedative Zolpidem, the anxiolytics Alpidem, Saridipem and Necopidem, the heart-failure drug Olprinone and the anti­ulcer drug Zolimidine (Baviskar *et al.*, 2011 ▸). As a continuation of our research on nitro­gen-bridgehead heterocycles (Tber *et al.*, 2015 ▸), we report herein on the mol­ecular and crystal structures, along with the Hirshfeld surface analysis, of the title compound, *N*-(*tert*-but­yl)-2-(phenyl­ethyn­yl)imidazo[1,2-*a*]pyridin-3-amine.
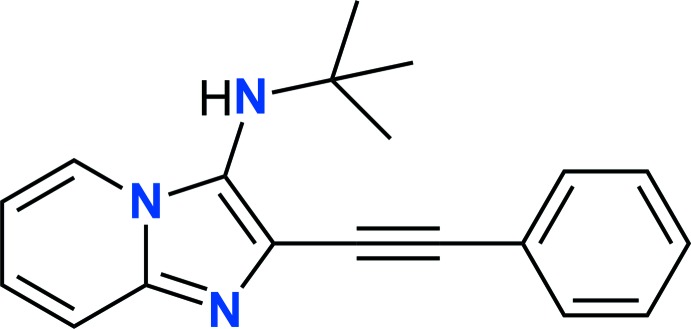


## Structural commentary   

**Figure 1:**
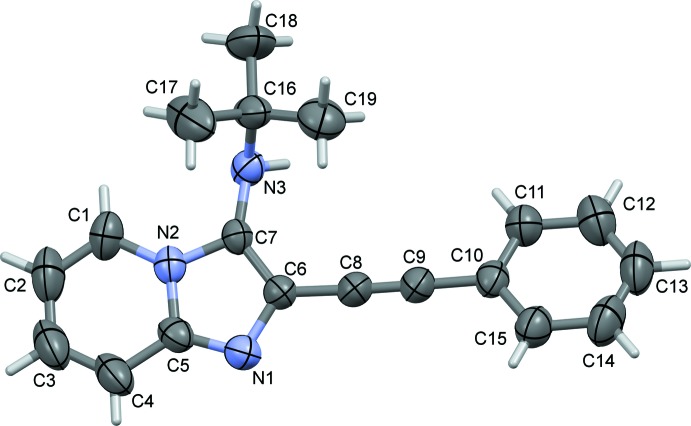
The mol­ecular structure of the title compound, with atom labelling. Displacement ellipsoids are drawn at the 50% probability level.

In the title compound (Fig. 1 ▸), the fused bicyclic imidazo[1,2-*a*]pyridine portion is slightly twisted with a dihedral angle of 3.6 (1)° between the mean planes of the five- and six-membered rings. The dihedral angle between the mean plane of the imidazo[1,2-*a*]pyridine moiety (r.m.s.deviation = 0.040 Å; N1/N2/C1–C7) and the phenyl ring (C10–C15) is 18.2 (1)°.

## Supra­molecular features   

**Figure 2:**
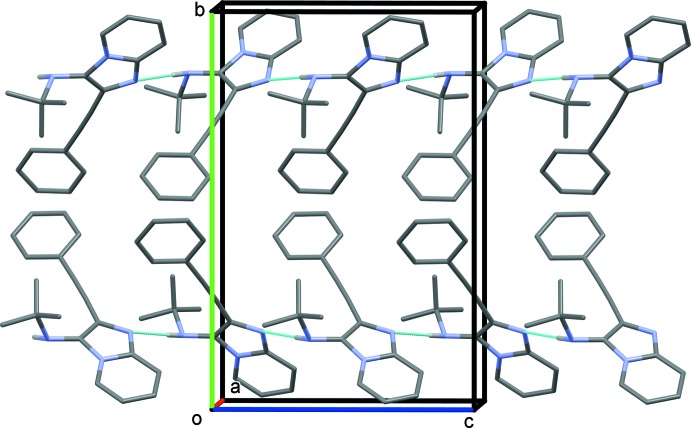
A partial view along the *a* axis of the crystal packing of the title compound, showing the N—H⋯N hydrogen-bonded chains (dashed lines; Table 1 ▸). The C-bound H atoms have been omitted.

**Figure 3:**
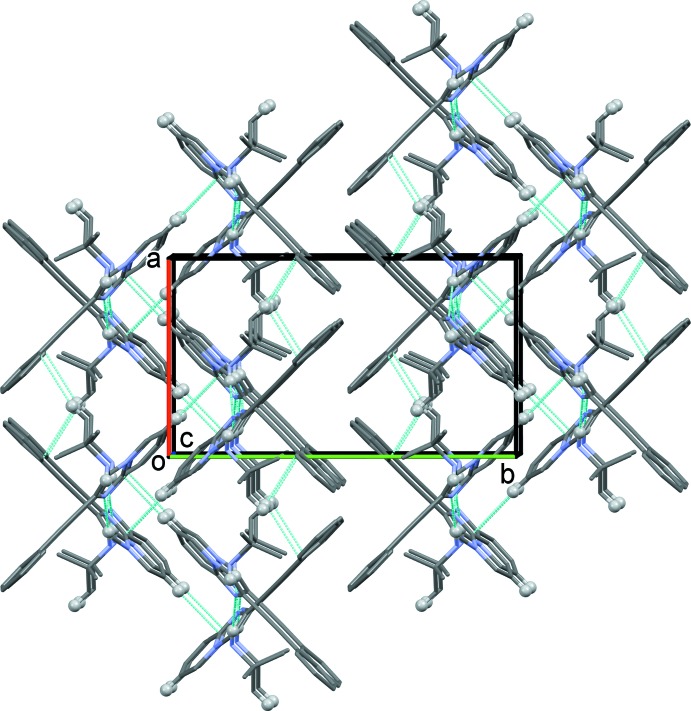
A view along the *c* axis of the crystal packing of the title compound. The C—H⋯π(ring) inter­actions and N—H⋯H hydrogen bonds (see Table 1 ▸) are indicated by dashed lines. Only the H atoms (grey balls) involved in the various inter­molecular inter­actions have been included.

In the crystal, mol­ecules are connected into chains along the *c*-axis direction by N3—H3*A*⋯N1^i^ hydrogen bonds (Table 1 ▸ and Fig. 2 ▸). These chains are linked by C2—H2⋯*Cg*4^ii^ and C17—H17*C*⋯*Cg*3^iii^ inter­actions, forming slabs parallel to the *ac* plane (Fig. 3 ▸ and Table 1 ▸).

## Hirshfeld surface analysis   

**Figure 4:**
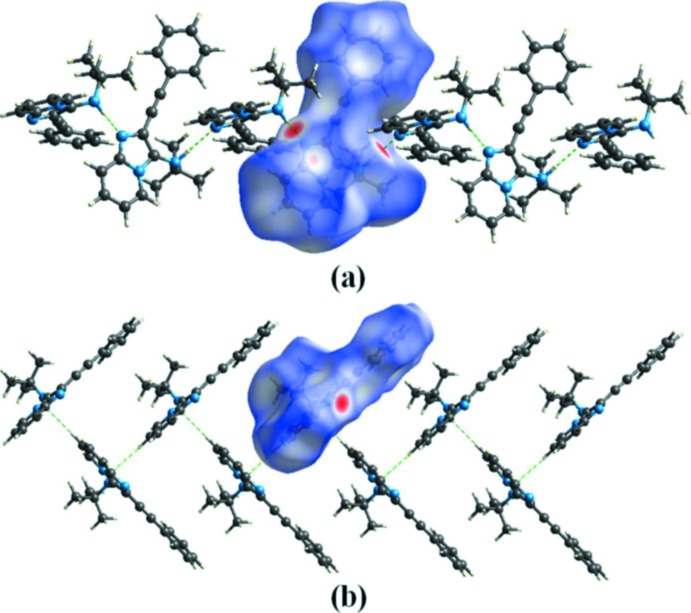
The Hirshfeld surface of the title compound mapped over *d*_norm_, showing (*a*) N—H⋯N hydrogen bonds and (*b*) C—H⋯π(ring) inter­actions.

**Figure 5:**
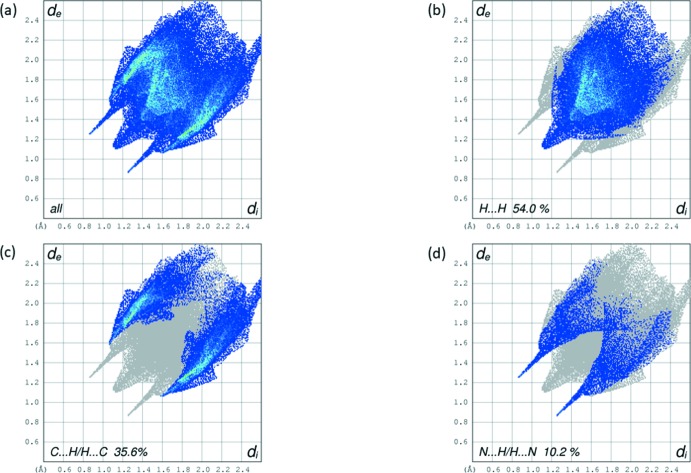
(*a*) The full two-dimensional fingerprint plot for the title compound and fingerprint plots delineated into (*b*) H⋯H, (*c*) C⋯H/H⋯C and (*d*) N⋯H/H⋯N contacts.

The Hirshfield surface analysis (Spackman & Jayatilaka, 2009 ▸; McKinnon *et al.*, 2007 ▸) was carried out using *CrystalExplorer17.5* (Turner *et al.*, 2017 ▸). The Hirshfeld surfaces and their associated two-dimensional fingerprint plots were used to qu­antify the various inter­molecular inter­actions in the title compound. The mol­ecular Hirshfeld surfaces were generated using a standard (high) surface resolution with the three-dimensional *d_norm_* surfaces mapped over a fixed colour scale of −0.379 (red) to 1.341 (blue). The red spots on the surface indicate the inter­molecular contacts involved in the hydrogen bonds. Fig. 4 ▸ *a* illustrates the inter­molecular N—H⋯N hydrogen bonding of the title compound with *d_norm_* mapped on Hirshfeld surface, and the C—H⋯π(ring) contacts are visualized in Fig. 4 ▸ *b*. The fingerprint plots are given in Fig. 5 ▸. They reveal that the principal inter­molecular inter­actions are H⋯H at 54.0% (Fig. 5 ▸ *b*) and C⋯H/H⋯C at 35.6% (Fig. 5 ▸ *c*), followed by N⋯H/H⋯N inter­actions at 10.2% (Fig. 5 ▸ *d*).

## Database survey   

A search of the Cambridge Structural Database (Version 5.40, last update May 2019; Groom *et al.*, 2016 ▸) for an imidazol[1,2-*a*]pyridine unit substituted with a phenyl­ethynyl group, *viz*. 2-(phenyl­ethyn­yl)imidazo[1,2-*a*]pyridine, gave zero hits. A search for *N*-(*tert*-but­yl)imidazo[1,2-*a*]pyridin-3-amines gave six hits (see supporting information). As in the title compound, the (*tert*-butyl-amine group lies almost normal to the plane of the imidazol[1,2-*a*]pyridine unit, with the torsion angle (*cf*. C16—N3—C7—C6; Fig. 1 ▸) varying from *ca* 75.0 to 90.7°, compared to −89.0 (2)° in the title compound.

## Synthesis and crystallization   

*tert*-Butyl­iso­nitrile (1.63 mmol, 1.05 equiv) was added to a mixture of 2-amino­pyridine (146 mg, 1.55 mmol), phenyl­propargyl aldehyde (1.63 mmol, 1.05 equiv) and perchloric acid (1 *M* solution in methanol, 0.07 mmol, 0.05 equiv) in a 50 ml flask at room temperature. The reaction mixture was stirred for 4 h at rt. The crude product was purified by flash chromatography on silica gel (2:8 ethyl acetate/petroleum ether). Colourless crystals were isolated when the solvent was allowed to evaporate (yield: 67%; m.p. 440–442 K).

## Refinement   

Crystal data, data collection and structure refinement details are summarized in Table 2 ▸. The C-bound H atoms were placed in idealized positions and refined as riding: C—H = 0.93–0.96 Å with *U*_iso_(H) = 1.5*U*_eq_(C-meth­yl) and 1.2*U*_eq_(C) for other C-bound H atoms. The NH H atom was located in a difference-Fourier map. Its parameters were adjusted to give N—H = 0.89 Å and it was then refined as riding with *U*_iso_(H) = 1.2*U*_eq_(N). The crystal studied was refined as an inversion twin, with a final BASF value of 0.3 (6).

## Supplementary Material

Crystal structure: contains datablock(s) global, I. DOI: 10.1107/S2056989019012751/su5507sup1.cif

Supporting information file. DOI: 10.1107/S2056989019012751/su5507Isup3.cdx

Structure factors: contains datablock(s) I. DOI: 10.1107/S2056989019012751/su5507Isup4.hkl

CSD search. DOI: 10.1107/S2056989019012751/su5507sup5.pdf

CCDC reference: 1953481

Additional supporting information:  crystallographic information; 3D view; checkCIF report

## Figures and Tables

**Table 1:** Hydrogen-bond geometry (Å, °) *Cg*3 and *Cg*4 are the centroids of the C10–C15 and N1/N2/C1–C7 rings, respectively.

*D*—H⋯*A*	*D*—H	H⋯*A*	*D*⋯*A*	*D*—H⋯*A*
N3—H3*A*⋯N1^i^	0.89	2.26	3.150 (2)	178
C2—H2⋯*Cg*4^ii^	0.93	2.98	3.890 (3)	167
C17—H17*C*⋯*Cg*3^iii^	0.96	2.95	3.896 (3)	170

Symmetry codes: (i) 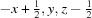; (ii) 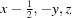; (iii) 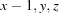.

**Table 2:** Experimental details

Crystal data	
Chemical formula	C_19_H_19_N_3_
*M*_r_	289.37
Crystal system, space group	Orthorhombic, *P* *c* *a*2_1_
Temperature (K)	298
*a*, *b*, *c* (Å)	9.3492 (2), 16.3716 (3), 10.8030 (2)
*V* (Å^3^)	1653.52 (6)
*Z*	4
Radiation type	Cu *K*α
μ (mm^−1^)	0.54
Crystal size (mm)	0.28 × 0.18 × 0.07

Data collection	
Diffractometer	Bruker D8 VENTURE PHOTON 100 CMOS
Absorption correction	Multi-scan (*SADABS*; Krause *et al.*, 2015 ▸)
*T*_min_, *T*_max_	0.86, 0.96
No. of measured, independent and observed [*I* > 2σ(*I*)] reflections	12219, 2789, 2610
*R*_int_	0.034
(sin θ/λ)_max_ (Å^−1^)	0.618

Refinement	
*R*[*F* ^2^ > 2σ(*F* ^2^)], *wR*(*F* ^2^), *S*	0.035, 0.094, 1.05
No. of reflections	2789
No. of parameters	204
No. of restraints	1
H-atom treatment	H-atom parameters constrained
Δρ_max_, Δρ_min_ (e Å^−3^)	0.12, −0.11

Computer programs: *APEX3* and *SAINT* (Bruker, 2016 ▸), *SHELXT* (Sheldrick, 2015*a* ▸), *Mercury* (Macrae, *et al.*, 2008 ▸), *SHELXL2018/1* (Sheldrick, 2015*b* ▸), *PLATON* (Spek, 2009 ▸) and *publCIF* (Westrip, 2010 ▸).

## supplementary crystallographic information

### Crystal data

**Table:**

C_19_H_19_N_3_	*D*_x_ = 1.162 Mg m^−^^3^
*M**_r_* = 289.37	Cu *K*α radiation, λ = 1.54178 Å
Orthorhombic, *P**c**a*2_1_	Cell parameters from 9967 reflections
*a* = 9.3492 (2) Å	θ = 2.7–72.4°
*b* = 16.3716 (3) Å	µ = 0.54 mm^−^^1^
*c* = 10.8030 (2) Å	*T* = 298 K
*V* = 1653.52 (6) Å^3^	Plate, colourless
*Z* = 4	0.28 × 0.18 × 0.07 mm
*F*(000) = 616	

### Data collection

**Table:**

Bruker D8 VENTURE PHOTON 100 CMOS diffractometer	2789 independent reflections
Radiation source: INCOATEC IµS micro-focus source	2610 reflections with *I* > 2σ(*I*)
Mirror monochromator	*R*_int_ = 0.034
ω scans	θ_max_ = 72.4°, θ_min_ = 2.7°
Absorption correction: multi-scan (*SADABS*; Krause *et al.*, 2015)	*h* = −11→10
*T*_min_ = 0.86, *T*_max_ = 0.96	*k* = −18→20
12219 measured reflections	*l* = −11→13

### Refinement

**Table:**

Refinement on *F*^2^	Secondary atom site location: difference Fourier map
Least-squares matrix: full	Hydrogen site location: mixed
*R*[*F*^2^ > 2σ(*F*^2^)] = 0.035	H-atom parameters constrained
*wR*(*F*^2^) = 0.094	*w* = 1/[σ^2^(*F*_o_^2^) + (0.0557*P*)^2^ + 0.0961*P*] where *P* = (*F*_o_^2^ + 2*F*_c_^2^)/3
*S* = 1.05	(Δ/σ)_max_ < 0.001
2789 reflections	Δρ_max_ = 0.12 e Å^−^^3^
204 parameters	Δρ_min_ = −0.11 e Å^−^^3^
1 restraint	Extinction correction: (*SHELXL-2018/1*; Sheldrick, 2015*b*), Fc^*^=kFc[1+0.001xFc^2^λ^3^/sin(2θ)]^-1/4^
Primary atom site location: structure-invariant direct methods	Extinction coefficient: 0.0082 (9)

### Special details

**Table:**

Geometry. All esds (except the esd in the dihedral angle between two l.s. planes) are estimated using the full covariance matrix. The cell esds are taken into account individually in the estimation of esds in distances, angles and torsion angles; correlations between esds in cell parameters are only used when they are defined by crystal symmetry. An approximate (isotropic) treatment of cell esds is used for estimating esds involving l.s. planes.
Refinement. Refinement of F^2^ against ALL reflections. The weighted R-factor wR and goodness of fit S are based on F^2^, conventional R-factors R are based on F, with F set to zero for negative F^2^. The threshold expression of F^2^ > 2sigma(F^2^) is used only for calculating R-factors(gt) etc. and is not relevant to the choice of reflections for refinement. R-factors based on F^2^ are statistically about twice as large as those based on F, and R- factors based on ALL data will be even larger. H-atoms attached to carbon were placed in calculated positions (C—H = 0.93 - 0.96 Å) while that attached to nitrogen was placed in a location derived from a difference map and its parameters adjusted to give N—H = 0.89 Å. All were included as riding contributions with isotropic displacement parameters 1.2 - 1.5 times those of the attached atoms. Refined as a 2-component inversion twin.

### Fractional atomic coordinates and isotropic or equivalent isotropic displacement parameters (Å^2^)

**Table:**

	*x*	*y*	*z*	*U*_iso_*/*U*_eq_	
N1	0.20226 (17)	0.18945 (10)	0.67968 (18)	0.0554 (4)	
N2	0.02701 (15)	0.12386 (9)	0.57937 (16)	0.0468 (4)	
N3	0.04415 (16)	0.17555 (10)	0.37051 (16)	0.0502 (4)	
H3A	0.116767	0.178516	0.317433	0.060*	
C1	−0.0790 (2)	0.06627 (13)	0.5650 (3)	0.0660 (6)	
H1	−0.121888	0.057890	0.488345	0.079*	
C2	−0.1189 (3)	0.02246 (16)	0.6643 (4)	0.0865 (9)	
H2	−0.188532	−0.017726	0.655677	0.104*	
C3	−0.0571 (3)	0.03642 (18)	0.7815 (3)	0.0880 (9)	
H3	−0.089464	0.007113	0.849685	0.106*	
C4	0.0493 (3)	0.09212 (17)	0.7959 (3)	0.0750 (7)	
H4	0.090129	0.101102	0.873222	0.090*	
C5	0.0964 (2)	0.13603 (12)	0.6914 (2)	0.0516 (4)	
C6	0.19772 (17)	0.21348 (11)	0.55737 (18)	0.0451 (4)	
C7	0.08982 (17)	0.17490 (10)	0.49231 (18)	0.0428 (4)	
C8	0.29559 (19)	0.27114 (12)	0.5063 (2)	0.0519 (4)	
C9	0.3769 (2)	0.31813 (12)	0.4592 (2)	0.0544 (5)	
C10	0.47560 (19)	0.37266 (12)	0.4002 (2)	0.0523 (5)	
C11	0.4920 (3)	0.37126 (14)	0.2731 (3)	0.0645 (6)	
H11	0.437975	0.335322	0.225549	0.077*	
C12	0.5885 (3)	0.42310 (16)	0.2164 (3)	0.0807 (8)	
H12	0.599034	0.421939	0.130835	0.097*	
C13	0.6684 (3)	0.47609 (16)	0.2855 (4)	0.0835 (9)	
H13	0.733481	0.510687	0.246809	0.100*	
C14	0.6530 (3)	0.47829 (15)	0.4107 (4)	0.0828 (8)	
H14	0.707257	0.514665	0.457238	0.099*	
C15	0.5569 (2)	0.42660 (13)	0.4694 (3)	0.0660 (6)	
H15	0.547037	0.428209	0.555024	0.079*	
C16	−0.0652 (2)	0.23805 (15)	0.3328 (2)	0.0611 (5)	
C17	−0.1897 (3)	0.2367 (2)	0.4234 (3)	0.0999 (11)	
H17A	−0.154972	0.247021	0.505559	0.150*	
H17B	−0.235099	0.184099	0.420959	0.150*	
H17C	−0.257708	0.278058	0.400960	0.150*	
C18	−0.1144 (4)	0.2142 (3)	0.2049 (3)	0.1049 (11)	
H18A	−0.160262	0.161741	0.208243	0.157*	
H18B	−0.033439	0.211409	0.150395	0.157*	
H18C	−0.180883	0.254133	0.174553	0.157*	
C19	0.0016 (3)	0.32261 (16)	0.3294 (3)	0.0822 (8)	
H19A	0.032518	0.337470	0.411127	0.123*	
H19B	−0.067847	0.361435	0.300697	0.123*	
H19C	0.082190	0.322349	0.274433	0.123*	

### Atomic displacement parameters (Å^2^)

**Table:**

	*U*^11^	*U*^22^	*U*^33^	*U*^12^	*U*^13^	*U*^23^
N1	0.0528 (9)	0.0651 (10)	0.0483 (9)	−0.0013 (7)	−0.0038 (7)	0.0078 (8)
N2	0.0442 (7)	0.0412 (7)	0.0549 (10)	−0.0007 (6)	0.0074 (6)	0.0037 (7)
N3	0.0454 (8)	0.0635 (9)	0.0418 (9)	−0.0071 (7)	0.0053 (6)	−0.0064 (7)
C1	0.0603 (11)	0.0552 (11)	0.0826 (18)	−0.0148 (9)	0.0118 (11)	−0.0021 (11)
C2	0.0792 (16)	0.0627 (14)	0.118 (3)	−0.0184 (12)	0.0232 (16)	0.0196 (16)
C3	0.0804 (16)	0.0857 (18)	0.098 (2)	0.0008 (13)	0.0196 (16)	0.0453 (17)
C4	0.0718 (14)	0.0842 (16)	0.0690 (17)	0.0079 (12)	0.0108 (12)	0.0342 (13)
C5	0.0512 (9)	0.0544 (10)	0.0493 (11)	0.0076 (8)	0.0024 (8)	0.0110 (9)
C6	0.0438 (8)	0.0480 (8)	0.0435 (10)	−0.0012 (7)	−0.0002 (7)	0.0028 (8)
C7	0.0409 (8)	0.0443 (8)	0.0433 (10)	−0.0013 (7)	0.0053 (7)	0.0016 (7)
C8	0.0472 (9)	0.0575 (10)	0.0509 (11)	−0.0078 (8)	−0.0058 (8)	0.0028 (9)
C9	0.0471 (9)	0.0565 (10)	0.0596 (12)	−0.0067 (8)	−0.0050 (9)	0.0053 (9)
C10	0.0427 (9)	0.0475 (9)	0.0668 (14)	−0.0015 (7)	−0.0020 (8)	0.0078 (9)
C11	0.0659 (12)	0.0624 (12)	0.0653 (15)	−0.0104 (10)	−0.0010 (11)	0.0067 (11)
C12	0.0838 (16)	0.0754 (15)	0.083 (2)	−0.0081 (14)	0.0188 (14)	0.0157 (14)
C13	0.0701 (14)	0.0645 (14)	0.116 (3)	−0.0156 (11)	0.0163 (15)	0.0214 (15)
C14	0.0692 (15)	0.0616 (13)	0.118 (3)	−0.0214 (11)	−0.0035 (16)	0.0017 (15)
C15	0.0618 (12)	0.0595 (11)	0.0767 (16)	−0.0109 (10)	−0.0044 (11)	0.0020 (12)
C16	0.0461 (9)	0.0900 (14)	0.0471 (11)	−0.0011 (10)	−0.0041 (8)	0.0094 (11)
C17	0.0589 (13)	0.153 (3)	0.088 (2)	0.0322 (16)	0.0195 (12)	0.039 (2)
C18	0.0932 (19)	0.163 (3)	0.0583 (16)	−0.032 (2)	−0.0257 (15)	0.013 (2)
C19	0.0817 (15)	0.0769 (15)	0.088 (2)	0.0111 (13)	−0.0097 (15)	0.0147 (16)

### Geometric parameters (Å, º)

**Table:**

N1—C5	1.327 (3)	C11—C12	1.381 (3)
N1—C6	1.379 (3)	C11—H11	0.9300
N2—C1	1.377 (2)	C12—C13	1.367 (4)
N2—C5	1.388 (3)	C12—H12	0.9300
N2—C7	1.388 (2)	C13—C14	1.361 (5)
N3—C7	1.383 (3)	C13—H13	0.9300
N3—C16	1.503 (3)	C14—C15	1.388 (3)
N3—H3A	0.8900	C14—H14	0.9300
C1—C2	1.344 (4)	C15—H15	0.9300
C1—H1	0.9300	C16—C18	1.508 (4)
C2—C3	1.410 (5)	C16—C19	1.519 (4)
C2—H2	0.9300	C16—C17	1.522 (3)
C3—C4	1.358 (4)	C17—H17A	0.9600
C3—H3	0.9300	C17—H17B	0.9600
C4—C5	1.409 (3)	C17—H17C	0.9600
C4—H4	0.9300	C18—H18A	0.9600
C6—C7	1.382 (2)	C18—H18B	0.9600
C6—C8	1.426 (2)	C18—H18C	0.9600
C8—C9	1.195 (3)	C19—H19A	0.9600
C9—C10	1.434 (3)	C19—H19B	0.9600
C10—C11	1.382 (3)	C19—H19C	0.9600
C10—C15	1.384 (3)		
			
C5—N1—C6	104.87 (17)	C13—C12—C11	120.4 (3)
C1—N2—C5	122.23 (19)	C13—C12—H12	119.8
C1—N2—C7	129.8 (2)	C11—C12—H12	119.8
C5—N2—C7	107.87 (15)	C14—C13—C12	120.1 (2)
C7—N3—C16	118.26 (16)	C14—C13—H13	120.0
C7—N3—H3A	112.2	C12—C13—H13	120.0
C16—N3—H3A	107.9	C13—C14—C15	120.4 (3)
C2—C1—N2	118.4 (3)	C13—C14—H14	119.8
C2—C1—H1	120.8	C15—C14—H14	119.8
N2—C1—H1	120.8	C10—C15—C14	119.8 (3)
C1—C2—C3	121.1 (2)	C10—C15—H15	120.1
C1—C2—H2	119.5	C14—C15—H15	120.1
C3—C2—H2	119.5	N3—C16—C18	106.2 (2)
C4—C3—C2	120.8 (2)	N3—C16—C19	110.32 (16)
C4—C3—H3	119.6	C18—C16—C19	109.9 (2)
C2—C3—H3	119.6	N3—C16—C17	109.6 (2)
C3—C4—C5	118.6 (3)	C18—C16—C17	110.6 (2)
C3—C4—H4	120.7	C19—C16—C17	110.1 (3)
C5—C4—H4	120.7	C16—C17—H17A	109.5
N1—C5—N2	111.09 (18)	C16—C17—H17B	109.5
N1—C5—C4	130.3 (2)	H17A—C17—H17B	109.5
N2—C5—C4	118.65 (19)	C16—C17—H17C	109.5
N1—C6—C7	112.28 (16)	H17A—C17—H17C	109.5
N1—C6—C8	122.66 (17)	H17B—C17—H17C	109.5
C7—C6—C8	125.06 (17)	C16—C18—H18A	109.5
C6—C7—N3	134.86 (17)	C16—C18—H18B	109.5
C6—C7—N2	103.85 (16)	H18A—C18—H18B	109.5
N3—C7—N2	121.22 (16)	C16—C18—H18C	109.5
C9—C8—C6	177.5 (2)	H18A—C18—H18C	109.5
C8—C9—C10	178.3 (2)	H18B—C18—H18C	109.5
C11—C10—C15	119.1 (2)	C16—C19—H19A	109.5
C11—C10—C9	120.2 (2)	C16—C19—H19B	109.5
C15—C10—C9	120.7 (2)	H19A—C19—H19B	109.5
C12—C11—C10	120.2 (3)	C16—C19—H19C	109.5
C12—C11—H11	119.9	H19A—C19—H19C	109.5
C10—C11—H11	119.9	H19B—C19—H19C	109.5
			
C5—N2—C1—C2	2.1 (3)	C8—C6—C7—N2	178.52 (17)
C7—N2—C1—C2	178.0 (2)	C16—N3—C7—C6	−89.0 (2)
N2—C1—C2—C3	1.8 (4)	C16—N3—C7—N2	94.6 (2)
C1—C2—C3—C4	−2.9 (5)	C1—N2—C7—C6	−174.58 (19)
C2—C3—C4—C5	0.2 (4)	C5—N2—C7—C6	1.78 (19)
C6—N1—C5—N2	1.5 (2)	C1—N2—C7—N3	2.8 (3)
C6—N1—C5—C4	−179.3 (2)	C5—N2—C7—N3	179.13 (16)
C1—N2—C5—N1	174.53 (18)	C15—C10—C11—C12	0.1 (4)
C7—N2—C5—N1	−2.2 (2)	C9—C10—C11—C12	−179.2 (2)
C1—N2—C5—C4	−4.8 (3)	C10—C11—C12—C13	0.0 (4)
C7—N2—C5—C4	178.52 (18)	C11—C12—C13—C14	−0.3 (4)
C3—C4—C5—N1	−175.7 (2)	C12—C13—C14—C15	0.4 (4)
C3—C4—C5—N2	3.5 (3)	C11—C10—C15—C14	0.1 (3)
C5—N1—C6—C7	−0.4 (2)	C9—C10—C15—C14	179.3 (2)
C5—N1—C6—C8	−179.81 (17)	C13—C14—C15—C10	−0.3 (4)
N1—C6—C7—N3	−177.7 (2)	C7—N3—C16—C18	−169.4 (2)
C8—C6—C7—N3	1.7 (3)	C7—N3—C16—C19	71.5 (2)
N1—C6—C7—N2	−0.9 (2)	C7—N3—C16—C17	−49.8 (3)

### Hydrogen-bond geometry (Å, º)

*Cg*3 and *Cg*4 are the centroids of the C10–C15 and N1/N2/C1–C7 rings, respectively.

**Table:**

*D*—H···*A*	*D*—H	H···*A*	*D*···*A*	*D*—H···*A*
N3—H3*A*···N1^i^	0.89	2.26	3.150 (2)	178
C2—H2···*Cg*4^ii^	0.93	2.98	3.890 (3)	167
C17—H17*C*···*Cg*3^iii^	0.96	2.95	3.896 (3)	170

Symmetry codes: (i) −*x*+1/2, *y*, *z*−1/2; (ii) *x*−1/2, −*y*, *z*; (iii) *x*−1, *y*, *z*.
