# Supplementary material for: Crystal structure and Hirshfeld surface analysis of N-(tert-but­yl)-2-(phenyl­ethyn­yl)imidazo[1,2-a]pyridin-3-amine
Source: Acta Crystallogr E Crystallogr Commun. 2019 Sep 27;75(Pt 10):1564–7. doi: 10.1107/S2056989019012751 (PMC6775750; doi:10.1107/S2056989019012751)

Search Overview

Search:	search1
Date/Time done:	Wed Jul 24 17:32:37 2019
Database(s):	CSD version 5.40 updates (Feb 2019)<br>CSD version 5.40 (November 2018)<br>CSD version 5.40 updates (May 2019)
Restriction Info:	No refcode restrictions applied
Filters:	None
Percentage Completed:	100%
Number of Hits:	10

Single query used. Search found structures that:

match

Query 1

Query 1

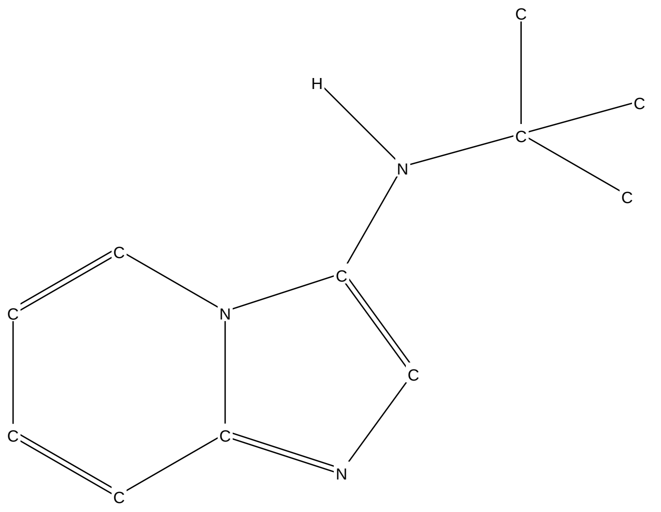

Search: search1 (Wed Jul 24 17:32:37 2019): Hits 1-4

PILGAV

Reference: R.Raja (2018)
CSD Communication(Private Communication) ,

Formula: $C_{17} H_{18} N_4 O_2$

Compound Name: N-t-butyl-2-(2-nitrophenyl)imidazo[1,2-a]pyridin-3-amine

Space Group:	Cc	***Cell:***	***a***	16.088(1)	***b***	22.045(1)	***c***	17.867(1)
Space Group No.:	9	***(Å,°)***	α	90.00	β	93.21(0)	γ	90.00

R-Factor (%): 4.46 ***Temperature(K):*** 296 ***Density(g/cm^3):*** 1.303

Parameters

Fragment 1
ANG1 (A) 67.565

Fragment 2
ANG1 (A) 70.298

Fragment 3
ANG1 (A) 70.351

Fragment 4
ANG1 (A) 70.605

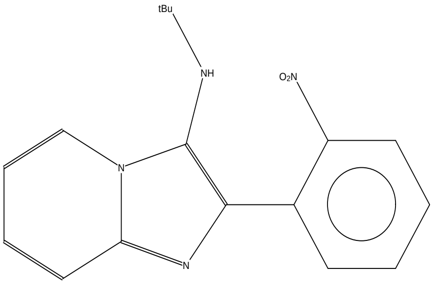

RIVTUO

Reference: G.Dhanalakshmi, M.Ramanjaneyulu, S.Thennarasu, S.Aravindhan (2018) *Acta Crystallogr.,Sect.E:Cryst.Commun.* ,**74,**1913

Formula: $C_{19} H_{23} N_3 O_1$

Compound Name: N-t-butyl-2-(4-methoxyphenyl)-5-methylimidazo[1,2-a]pyridin-3-amine

Space Group:	P21/c	***Cell:***	***a***	9.236(0)	***b***	15.639(1)	***c***	11.984(1)
Space Group No.:	14	***(Å,°)***	α	90.00	β	94.00(0)	γ	90.00

R-Factor (%): 4.66 ***Temperature(K):*** 296 ***Density(g/cm^3):*** 1.190

Parameters

Fragment 1
ANG1 (A) 79.343

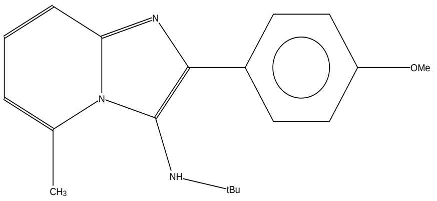

RIVVAW

Reference: G.Dhanalakshmi, M.Ramanjaneyulu, S.Thennarasu, S.Aravindhan (2018) *Acta Crystallogr.,Sect.E:Cryst.Commun.* ,**74,**1913

Formula: $C_{19} H_{24} N_4$

Compound Name: N-t-butyl-2-(4-(dimethylamino)phenyl)imidazo[1,2-a]pyridin-3-amine

Space Group:	C2/c	***Cell:***	***a***	34.919(1)	***b***	8.466(0)	***c***	11.836(0)
Space Group No.:	15	***(Å,°)***	α	90.00	β	91.06(0)	γ	90.00

R-Factor (%): 4.92 ***Temperature(K):*** 296 ***Density(g/cm^3):*** 1.171

Parameters

Fragment 1
ANG1 (A) 82.762

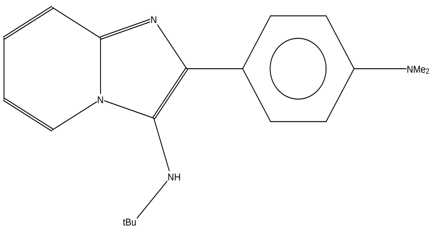

AYEWOS

Reference: A.Sagar, V.N.Babu, A.H.Shinde, D.S.Sharada (2016)
Org.Biomol.Chem. ,**14,**10366

Formula: $C_{19} H_{22} N_3{}^{1+}, Br_1{}^{1-}$

Compound Name: 13-(t-butylamino)-5,6-dihydropyrido[2',1':2,3]imidazo[5,1-a]isoquinolin-7-ium bromide

Space Group:	P-1	***Cell:***	***a***	9.867(0)	***b***	10.295(0)	***c***	11.035(0)
Space Group No.:	2	***(Å,°)***	α	117.59(0)	β	105.75(0)	γ	97.85(0)

R-Factor (%): 3.54 ***Temperature(K):*** 300 ***Density(g/cm^3):*** 1.359

Parameters

Fragment 1
ANG1 (A) 52.785

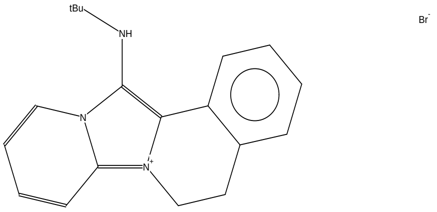

Search: search1 (Wed Jul 24 17:32:37 2019): Hits 5-8

IBUSAC

Reference: Jean Dam, Z.Ismail, T.Kurebwa, Nadia Gangat, L.Harmse, H.M.Marques, A.Lemmerer, M.L.Bode, C.B.de Koning (2017) *Eur.J.Med.Chem.* ,**126,**353

Formula: C_{22} H_{23} Br_1 N_4

Compound Name: 6-bromo-2-(pyridin-2-yl)-N-(tricyclo[3.3.1.1^{3,7}]decan-1-yl)imidazo[1,2-a]pyridin-3-amine

Space Group:	P21/c	***Cell:***	***a***	12.326(1)	***b***	7.534(1)	***c***	20.376(3)
Space Group No.:	14	***(Å,°)***	α	90.00	β	95.31(1)	γ	90.00

R-Factor (%): 7.25 ***Temperature(K):*** 173 ***Density(g/cm^3):*** 1.493

Parameters

Fragment 1
ANG1 (A) 59.889

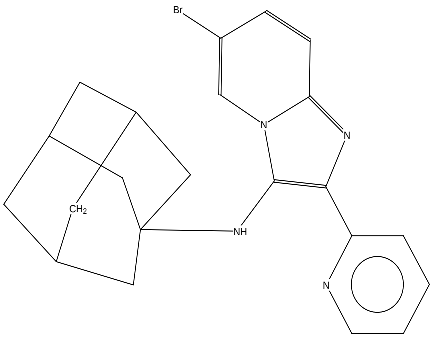

IBUTAD

Reference: Jean Dam, Z.Ismail, T.Kurebwa, Nadia Gangat, L.Harmse, H.M.Marques, A.Lemmerer, M.L.Bode, C.B.de Koning (2017) *Eur.J.Med.Chem.* ,**126,**353

Formula: C_{26} H_{29} Br_1 N_4 O_4 Zn_1

Compound Name: bis(acetato)-(6-bromo-2-(pyridin-2-yl)-N-(tricyclo[3.3.1.1^{3,7}]decan-1-yl) imidazo[1,2-a]pyridin-3-amine)-zinc(ii)

Space Group:	P21/c	***Cell:***	***a***	13.170(1)	***b***	14.741(1)	***c***	17.228(1)
Space Group No.:	14	***(Å,°)***	α	90.00	β	129.43(0)	γ	90.00

R-Factor (%): 8.69 ***Temperature(K):*** 173 ***Density(g/cm^3):*** 1.560

Parameters

Fragment 1
ANG1 (A) 57.522

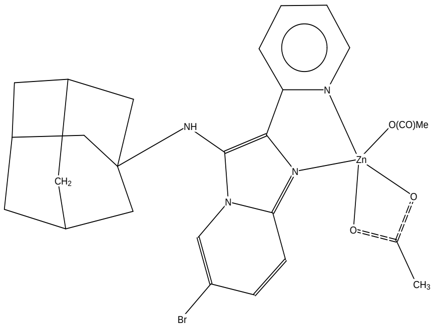

IBUVAF

Reference: Jean Dam, Z.Ismail, T.Kurebwa, Nadia Gangat, L.Harmse, H.M.Marques, A.Lemmerer, M.L.Bode, C.B.de Koning (2017) *Eur.J.Med.Chem.* ,**126,**353

Formula: C_{26} H_{29} Br_1 Cu_1 N_4 O_4,2(C_1 H_4 O_1)

Compound Name: bis(acetato)-(6-bromo-2-(pyridin-2-yl)-N-(tricyclo[3.3.1.1^{3,7}]decan-1-yl) imidazo[1,2-a]pyridin-3-amine)-copper(ii) methanol solvate

Space Group:	P-1	***Cell:***	***a***	9.825(0)	***b***	11.144(0)	***c***	13.779(1)
Space Group No.:	2	***(Å,°)***	α	76.23(0)	β	82.81(0)	γ	85.64(0)

R-Factor (%): 3.31 ***Temperature(K):*** 173 ***Density(g/cm^3):*** 1.530

Parameters

Fragment 1
ANG1 (A) 87.787

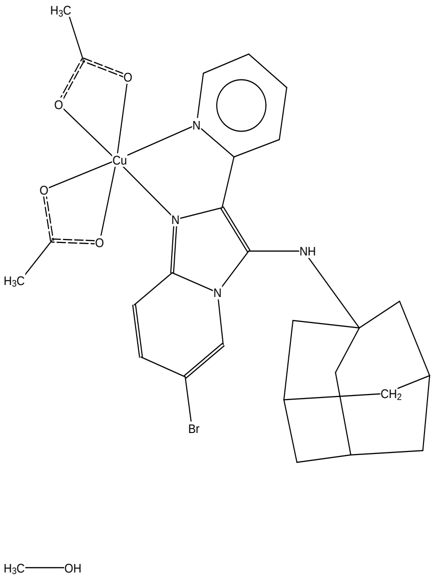

NOGRIM

Reference: G.Marandi, L.Saghatforoush, R.Mendoza-Merono, S.Garcia-Granda (2014) *Tetrahedron Lett.* ,**55,**3052

Formula: C_{18} H_{18} N_4 O_4

Compound Name: 3-(t-Butylamino)-2-(3-nitrophenyl)imidazo[1,2-a]pyridine-8-carboxylic acid

Space Group:	P-1	***Cell:***	***a***	8.401(0)	***b***	10.213(0)	***c***	10.714(0)
Space Group No.:	2	***(Å,°)***	α	94.78(0)	β	112.84(0)	γ	97.01(0)

R-Factor (%): 5.86 ***Temperature(K):*** 293 ***Density(g/cm^3):*** 1.414

Parameters

Fragment 1
ANG1 (A) 83.632

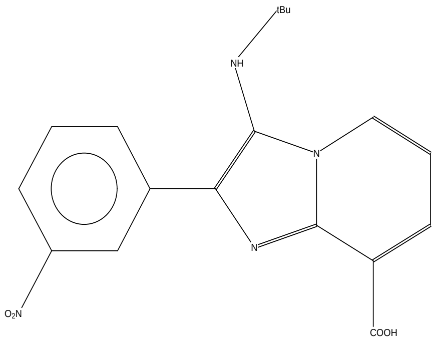

Search: search1 (Wed Jul 24 17:32:37 2019): Hits 9-10

SOZJEY

Reference: K.G.Kishore, U.M.V.Basavanag, A.Islas-Jacome, R.Gamez-Montano (2015) *Tetrahedron Lett.* ,**56,**155

Formula: $C_{20} H_{19} N_3 O_2$

Compound Name: 3-(3-(t-butylamino)imidazo[1,2-a]pyridin-2-yl)-4H-chromen-4-one

Space Group:	P21/c	***Cell:***	***a*** 14.185(0)	***b*** 6.766(0)	***c*** 17.856(0)
Space Group No.:	14	***(Å,°)***	α 90.00	β 99.58(0)	γ 90.00

R-Factor (%): 4.40 ***Temperature(K):*** 298 ***Density(g/cm^3):*** 1.311

Parameters

Fragment 1
ANG1 (A) 58.934

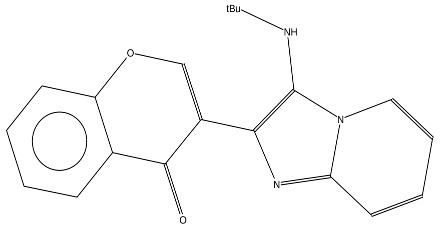

VAJRIK

Reference: P.Manvar, F.Shaikh, R.Kakadiya, K.Mehariya, Ranjan Khunt, B.Pandey, Anamik Shah (2016) *Tetrahedron* ,**72,**1293

Formula: $C_{21} H_{21} N_3 O_3, C_1 H_1 Cl_3$

Compound Name: 3-(3-(t-butylamino)-7-methylimidazo[1,2-a]pyridin-2-yl)-4-hydroxy-2H-chromen-2-one chloroform solvate

Space Group:	P-1	***Cell:***	***a*** 8.407(0)	***b*** 11.711(0)	***c*** 12.066(0)
Space Group No.:	2	***(Å,°)***	α 88.93(0)	β 82.32(0)	γ 81.08(0)

R-Factor (%): 7.86 ***Temperature(K):*** 293 ***Density(g/cm^3):*** 1.379

Parameters

Fragment 1
ANG1 (A) 83.254

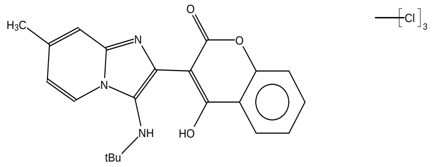

Supplement: Supplementary file 4 [file e-75-01564-sup5.pdf]
